# 2D MXene Ti_3_C_2_T_*x*_ nanosheets in the development of a mechanically enhanced and efficient antibacterial dental resin composite

**DOI:** 10.3389/fchem.2022.1090905

**Published:** 2022-12-16

**Authors:** Yingzi Hu, Zhiliang Xu, Junmei Pu, Lanping Hu, You Zi, Mengke Wang, Xingmei Feng, Weichun Huang

**Affiliations:** ^1^ Department of Stomatology, Affiliated Hospital of Nantong University, Nantong, China; ^2^ School of Chemistry and Chemical Engineering, Nantong University, Nantong, China

**Keywords:** MXene, Ti_3_C_2_T_x_, nanocomposite, antibacterial, dental resin

## Abstract

The bacterial accumulation at the margins of dental resin composites is a main cause of secondary caries, which may further lead to prosthodontic failure. In this regard, this study for the first time incorporated 2D MXene Ti_3_C_2_T_x_ nanosheets (NSs) into epoxy resin at different mass ratios (0, 0.5, 1.0, and 2.0 wt%) by solution blending and direct curing for dental applications. Compared to the pure resin, the as-fabricated MXene/resin composite not only exhibited improved mechanical and abrasive results but also displayed gradually improved antibacterial activity with MXene loading which was further enhanced by illumination in natural light due to the high photothermal efficiency of MXene. In addition, the cytotoxicity result demonstrated that the MXene-modified resin did not cause severe damage to normal cells. This novel MXene/resin nanocomposite could pave the way for new designs for high-performance, multifunctional nanocomposites to effectively protect dental health in daily life.

## Introduction

Owing to their excellent aesthetic and physiochemical properties, resin composites, composed of a polymerizable resin matrix, silanized inorganic fillers, and initiator systems, have been extensively utilized to restore various dental defects (Wang Y. et al., 2021). Although resin composites have shown great progress, secondary caries mainly due to high bacterial activity in the marginal space between the restorative material and the dental tissue is among the most common reasons for restoration replacement. Many efforts have been made to address this challenge, including i) the development of materials with good anti-cariogenic properties by releasing active chemicals such as Ca^2+^ and PO_4_^3−^ (Yang et al., 2021a), and chlorhexidine (Boaro et al., 2019; Campos et al., 2020), which can effectively inhibit bacterial growth and weaken colonization and acid production on the tooth surface, and ii) the direct incorporation of antibacterial agents such as N-doped TiO_2_ (Ahmad Fauzi et al., 2022), Ag nanoparticles (NPs) (Yang et al., 2021b), and Ca-doped SiO_2_ NPs (Zhang et al., 2018), into dental resin composites. These alternatives have exhibited high antibacterial efficiency in both planktonic and biofilm form; however, current antibacterial technologies still encounter two major limitations, namely, i) reduced mechanical performance due to the introduction of insoluble materials, and ii) the short duration of the antibacterial activity owing to the exhaustion of antibacterial agent release, which greatly limits the practical applications of these materials. For example, the incorporation of unmodified fillers (e.g., SiO_2_ NPs) with a resin matrix led to the poor mechanical performance of dental composites due to the weak interfacial bonding between the resin and filler (Kim et al., 2007; Karabela and Sideridou, 2008). Furthermore, soluble Ca^2+^ and PO_4_^3−^, and Ag^+^ showed rapid release rates, which largely restricted their efficiency in prolonged use (Mizerska et al., 2018; Sa et al., 2018). Therefore, a chemically stable, mechanically reinforced material with a long-lasting antibacterial capability is urgently needed for dental composites.

MXenes have drawn research attention since the successful synthesis of MXene Ti_3_C_2_T_*x*_ nanosheets (NSs) by a selective etching method in 2011 (Naguib et al., 2011). Subsequent research on MXenes has resulted in the systematic development and study of >50 different MAX phases and >30 MXenes (Verger et al., 2019; Jiang et al., 2020; Wang et al., 2020). The general formula of MXenes is M_*n*+1_X_*n*_T_*x*_ (*n* = 1, 2, 3, or 4), in which M is an early transition metal (such as Ti, V, Cr, Nb, and Hf), X is carbon and/or nitrogen, and T_*x*_ represents terminal groups (such as -F, and-OH) derived from the synthesis procedures (Chen et al., 2020; Gao et al., 2020). MXene as a layered 2D material has been used in various applications, including non-linear photonics (Jiang et al., 2018; Huang et al., 2020a; Ma et al., 2020) and photocatalysis (Wang et al., 2018; Prasad et al., 2020), due to their remarkable optoelectronic and optical properties. In addition, Zhang et al. (2019) demonstrated that the strength and toughness of MXene/Kevlar nanofiber composite membranes can reach up to ∼101 MPa and 2.64 MJ m^−3^, respectively, comparable to natural nacre (∼130 MPa and 1.9 MJ m^−3^) (Wan et al., 2015). Moreover, the Young’s modulus achieved a considerably high value of ∼3.4 GP. Li et al. (2017) reported an estimated photothermal conversion efficiency of MXene Ti_3_C_2_T_*x*_ NSs of ∼100%, indicating that MXene Ti_3_C_2_T_*x*_ NSs is an ideal candidate for energy conversion. Matthews et al. (2021) demonstrated that delaminated MXene NSs can not only be stored in suspension for several months without degradation but can also be easily re-dispersed and processed into films. The combination of these MXene improvements can alleviate existing challenges faced by the community and allow for their wider use in multifunctional applications.

The present study describes for the first time the incorporation of MXene Ti_3_C_2_T_*x*_ NSs as functional fillers in dental epoxy resin. MXene Ti_3_C_2_T_*x*_ NSs are conducive to creating a micromechanical domain between filler/matrix resin to enhance the mechanical and wear properties of composites. Moreover, the as-fabricated MXene/resin composites showed progressively enhanced antibacterial activity with MXene loading, which is further improved under illumination due to the high photothermal efficiency of MXene. Furthermore, the introduction of MXene into dental resin showed no apparent effects on cytotoxicity and stability, suggesting the promise of this kind of MXene/resin for dental applications. These results provide a fundamental study of the properties of MXenes and greatly expand their range of potential applications.

## Experimental section

### Fabrication of MXene Ti_3_C_2_T_*x*_ NSs

MXene Ti_3_C_2_T_x_ *NSs* were synthesized according to a previously reported hydrofluoric acid (HF) etching method (Naguib et al., 2011; Huang et al., 2020a). The raw Ti_3_AlC_2_ powder was etched in the HF solution with a Ti_3_AlC_2_ concentration of 0.1 g ml^−1^. The etching reaction was conducted in an open plastic beaker at 40°C for 48 h with continuous stirring. After selective removal of the Al layer, the obtained mixture was separated at a centrifugation speed of 5,000 rpm for 25 min; the resultant precipitate was then re-dispersed into deionized water. The dispersion was filtered through a hydrophilic 200 nm polytetrafluoroethylene porous membrane. The etched Ti_3_AlC_2_ was sufficiently washed with ethanol and deionized water, respectively, and dried in a vacuum oven at 80°C overnight. Next, 2.0 g of the as-etched Ti_3_AlC_2_ was added to 400 ml deionized water, and the mixture was sonicated for 2 h before centrifugation at 6,000 rpm for 30 min. Finally, the supernatant containing ultrathin 2D Ti_3_C_2_T_x_ NSs was collected by centrifugation at 18,000 rpm for 30 min and dried in a vacuum oven at 60°C overnight.

### Preparation of MXene/resin nanocomposites

The resin monomer ethoxylated bisphenol A dimethacrylate (BisGMA, Sigma-Aldrich Co., St Louis, MO, United States) and monomer triethyleneglycol dimethacrylate (TEGDMA, Sigma-Aldrich Co., St Louis, MO, United States) were mixed at a mass ratio of 7:3. A predetermined amount of MXene Ti_3_C_2_T_*x*_ NSs was added to the BisGMA/TEGDMA mixture with 4–6 ml of ethanol. Afterward, the as-obtained mixture was placed into the ultrasonic constant temperature cleaner for 1 h to obtain a well-dispersed suspension. The suspension was then heated at 80°C for 8 h to completely evaporate the ethanol. The silanized SiO_2_ nanoparticles (NPs) (Aladdin Holdings Group Co., Ltd., China) with a mass ratio of 70%, camphorquinone (CQ, Aladdin Holdings Group Co., Ltd., China) as the visible light polymerization initiator with a mass ratio of 1%, and N,N-dimethylaminoethyl methacrylate (DMAEMA, Sigma-Aldrich Co., St Louis, MO, United States) with a mass ratio of 2% were added to the MXene Ti_3_C_2_T_*x*_ NS/resin matrix. The resulting composite was then cured with a LED blue light curing lamp of 3 W (*λ* = 420–480 nm, Guilin Weirun Medical Technology Co., LTD., China) for 2 min. MXene Ti_3_C_2_T_*x*_ NSs concentrations of 0, 0.5, 1.0, and 2.0 wt%) were used. The corresponding samples were abbreviated as pure resin, 0.5 wt% MXene/resin, 1.0 wt% MXene/resin, and 2.0 wt% MXene/resin, respectively.

### Structural characterization

The morphology and dimension of the as-synthesized MXene Ti_3_C_2_T_x_ NSs were determined by both scanning electron microscopy (SEM, JSM-6701F, JEOL) and transmission electron microscopy (TEM, FEI Tecnai G2 F30). High-resolution transmission electron microscopy (HRTEM) was also conducted to determine the atomic arrangement. Energy-dispersive X-ray spectroscopy (EDS) analysis was obtained using an FEI Tecnai G2 F30 TEM equipped with the Oxford EDAX EDS system. X-ray diffraction (XRD) analysis was performed on an X’Pert-Pro MPD diffractometer with a Cu K-α radiation source at room temperature. Ultraviolet-visible-near infrared light (UV-Vis-NIR) absorption spectroscopy was recorded in the spectral range of 200–1000 nm using a UV-vis absorbance spectrometer (Cary 60, Agilent). The mechanical properties of the pure resin and MXene/resin nanocomposites were characterized by tensile testing. All tensile tests were performed using an Instron 3365 machine with a crosshead speed of 1.0 mm min^−1^ at room temperature. A standard setup was used for friction using an established procedure with a pin-on-disk tribometer with rotating motion. The sample size was 30.0 mm (length) × 7.0 mm (width) × 4.0 mm (height). The pin was fixed on a stationary holder with an applied load of 5.0 N. The friction forces were measured at room temperature with semiconductor strain gauges that were then digitized and collected on a personal computer. The reported values represented the averages of at least three measurements. Thermogravimetric analysis (TGA) was performed with a heating rate of 10°C min^−1^ from room temperature to 800°C under a continuous N_2_ flow. The temperature of thermal degradation (*T*_d_) was measured at the point of 5 wt% loss relative to the weight at room temperature. Differential scanning calorimetry (DSC) was performed on a TA Q2000 instrument under an N_2_ atmosphere from −60°C to 200°C at heating and cooling rates of 10°C min^−1^. The first cooling and the second heating scans were used to determine the glass transition temperature (*T*_g_).

### Antibacterial activity evaluation

To evaluate the efficiency of the as-fabricated MXene/resin nanocomposite against cariogenic bacteria, direct contact tests were performed using *S. mutans*. The formation of dental plaque biofilm can be divided into three basic stages: i) acquired biofilm formation; ii) bacterial adhesion and copolymerization; and iii) mature plaque biofilm. Plaque was stained by a plaque display agent after 12 h. To explore the inhibition of materials on the early growth and reproduction of bacteria, different incubation times (12 and 24 h) were used. MXene/resin nanocomposites with different concentrations of MXene Ti_3_C_2_T_x_ NSs (0, 0.5, 1.0, and 2.0 wt%) were cut into discs 8 mm in diameter and 0.5 mm thick. Afterward, the discs were immersed in deionized water for 1 h to remove free Ti_3_C_2_T_x_ NSs on the surface. After sterilization by ultraviolet illumination for 2 h, a 10 μL suspension containing *S. mutans* (∼10^6^ CFU mL^−1^) was poured onto the surface of each disc. For better contact between the MXene/resin nanocomposite and the suspension, a polyethylene film was used to gently cover the suspension surface on the disc. After anaerobic incubation for 12 h, both the discs and polyethylene (PE) film were placed in a test tube containing 10 ml of axenic physiological saline, from which the bacteria were completely eluted by shaking and then collected. Subsequently, 100 μL of the diluted eluent was transferred to an agar plate for 12-h anaerobic incubation. The groups with 30–300 bacterial colonies in each gradient were chosen to calculate the quantity of original live bacteria on the discs. The blank group was also set under the same conditions in the absence of the MXene/resin nanocomposite. All experiments were repeated at least three times. The resin discs of the above-mentioned direct contact experiment were soaked in a 2.5% glutaraldehyde fixative solution for 24 h. Subsequently, the impurities and residual fixative solution were completely washed with phosphate buffer solution and the resin disc was dehydrated in gradients of 30, 50, 70, 90, and 100% ethanol for 15 min at each concentration, respectively. The obtained sample was dried for SEM characterization.

### Cytotoxicity analysis

Cell viability on contact with the surface of the MXene/resin nanocomposite was also assessed. Cell viability and proliferation were tested using Cell Counting Kit-8 (CCK-8, Beyotime, China) according to the manufacturer’s instructions. A total of 10 µL CCK-8 was added per well in 96-well plates and incubated at 37°C for 2 h. The optical density was measured at 450 nm to determine the cell survival rate of each group using the following equation: cell viability = (OD_e_−OD_b_)/(OD_c_−OD_b_), where OD_e_, OD_b_, and OD_c_ denote the OD values of the experimental, blank, and control groups, respectively.

## Results and discussion

**FIGURE 1:**
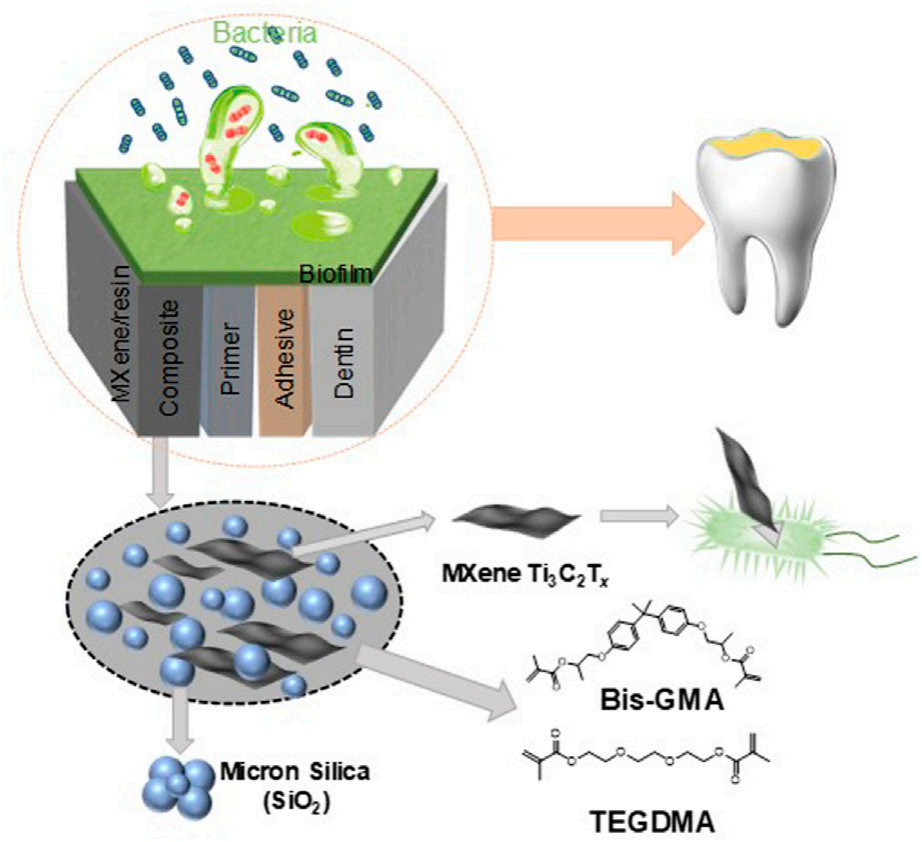
Schematic diagram of the process for the fabrication of the MXene/resin nanocomposites.

**FIGURE 2:**
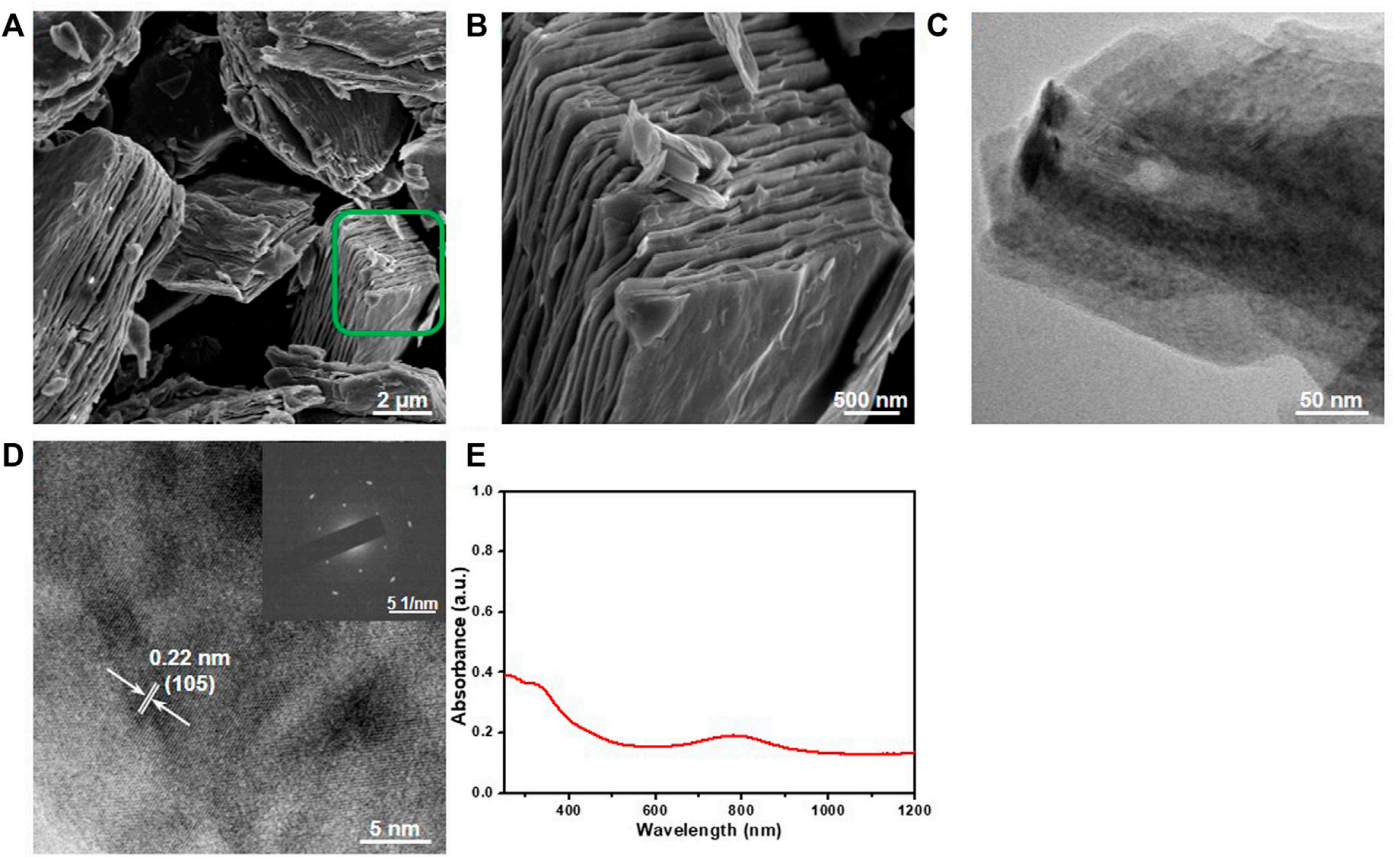
Structural characterization of the as-synthesized MXene Ti_3_C_2_T_*x*_ NSs. **(A)** SEM image of the as-etched Ti_3_AlC_2_ and **(B)** the enlarged area in **(A)**. **(C)** TEM image of the as-exfoliated MXene Ti_3_C_2_T_*x*_ NSs. **(D)** HRTEM image of the as-exfoliated MXene Ti_3_C_2_T_*x*_ NSs; inset: SAED pattern. **(E)** UV-Vis-NIR spectroscopy of the as-exfoliated MXene Ti_3_C_2_T_*x*_ NSs.

The schematic diagram of the process for the fabrication of the MXene/resin nanocomposites for antibacterial dental applications is presented in Figure 1. The structural characterization of the as-etched Ti_3_Al_2_C and as-exfoliated MXene Ti_3_C_2_T_x_ NSs are shown in Figure 2. The SEM images in Figures 2A, B show that the basal planes fan out and spread apart after HF treatment, demonstrating the successful removal of Al from Ti_3_AlC_2_. The TEM image of the exfoliated NSs (Figure 2C) shows that the as-exfoliated MXene Ti_3_C_2_T_x_ NSs is quite thin due to higher transparency compared to a super-thin carbon film. The lateral size of the as-exfoliated MXene Ti_3_C_2_T_x_ NSs was around 300 nm. The crystal structure of the as-exfoliated MXene Ti_3_C_2_T_x_ NSs was also confirmed by HRTEM (Figure 2D) and showed good agreement with previous reports (Naguib et al., 2011; Wei et al., 2021). Figure 2E shows the absorption spectrum of the MXene Ti_3_C_2_T_x_ NSs, ranging from 250 nm to 12,00 nm, indicating the broadband absorption of the as-fabricated MXene Ti_3_C_2_T_x_ NSs.

**FIGURE 3:**
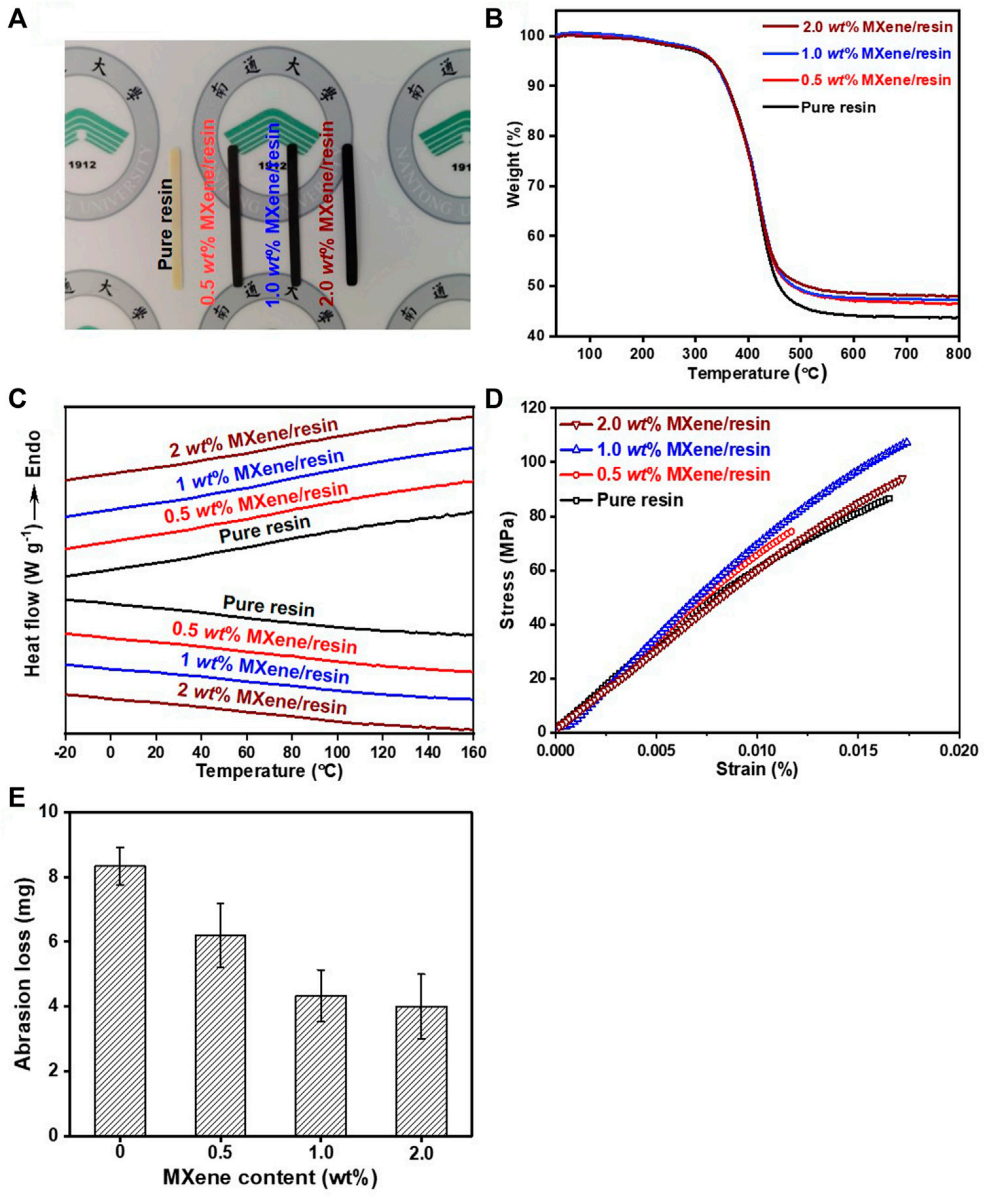
**(A)** Optical images of as-synthesized MXene/resin nanocomposites with different weight percentages of MXene Ti_3_C_2_T_*x*_ NSs. **(B)** DSC and **(C)** TGA curves of the MXene/resin nanocomposites. **(D)** Mechanical properties and **(E)** abrasion loss as a function of MXene content in the resin.

Figure 3 shows the structural characterization of the pure resin and MXene/resin nanocomposites with different loadings of MXene Ti_3_C_2_T_x_ NSs. The optical specimen pictures of the as-obtained pure resin and MXene/resin nanocomposites are shown in Figure 3A, in which the colors of the as-fabricated specimens gradually darkened with increasing MXene Ti_3_C_2_T_x_ NSs concentrations due to the nature of the black MXenes Ti_3_C_2_T_x_ NSs. The TGA curves in Figure 3B showed that all the samples had T_d_ of ∼333 °C, similar to the previous reports (Garcia et al., 2019), indicating that the incorporation of MXene Ti_3_C_2_T_x_ NSs in the studied loadings had negligible effects on the thermal stability. Notably, the residual weight percentages for all the tested samples at 800°C increased with increased loading of MXene Ti_3_C_2_T_x_ NSs with residual weight percentages for the pure resin, 0.5 wt% MXene/resin, 1.0 wt% MXene/resin, and 2.0 wt% MXene/resin, of 43.8, 46.5, 47.2, and 48.0 wt%, respectively*,* which was attributed to the higher thermal stability of the pure MXene Ti_3_C_2_T_x_ NSs (Levitt et al., 2019; Cao et al., 2018). Moreover, the DSC results of the pure resin and MXene/resin nanocomposites with different loadings showed no heat flow change during the heating/cooling scans (Figure 3C), similar to those of eugenyl methacrylate/Bis-GMA/TEGDMA resin (Yu et al., 2015) and boron nitride NSs/resin nanocomposites (Yu et al., 2016; Chen et al., 2019). The mechanical properties of the pure resin and MXene/resin nanocomposites were measured by tensile tests, as shown in Figure 3D. The strain-at-break of all the studied specimens did not change remarkably with increasing MXene loading, while the tensile strength increased in the MXene loading range of 0–1.0 wt% and then declined, which could be attributed to severe aggregation at a relatively high MXene loading, similar to the MXene Ti_3_C_2_T_*x*_ NS/aramid nanofiber composite paper (Xie et al., 2019) and MXene Ti_3_C_2_T_*x*_ NS/bacterial cellulose composite paper (Jiao et al., 2019). With increasing MXene Ti_3_C_2_T_*x*_ NS loading, the abrasion loss of the MXene/resin samples gradually decreased (Figure 3E), similar to that of SiO_2_ NP/epoxy resin (Alam et al., 2020) and SiO_2_ NP/polyurethane (Malaki et al., 2018) composites.

**FIGURE 4:**
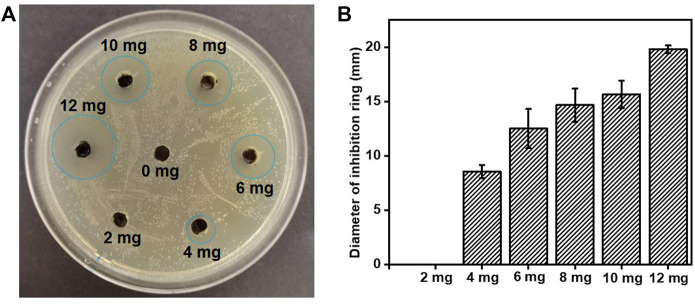
**(A)** Antibacterial activity of MXene Ti_3_C_2_T_*x*_ NSs against *S. mutans*; the edges of the inhibition rings were marked in ocean blue cycles. **(B)** Inhibition ring diameter.

The inhibition zone approach was used to determine the antibacterial activity based on the size of a clear circle formed by inhibiting the growth of bacteria surrounding the tested samples in agar plates (Wu et al., 2020). As shown in Figures 4A, B, except for the group with 0 mg or 2 mg MXene, other groups showed superior antibacterial activity against *S. mutans*. The lack of antibacterial activity for the group with 0 mg MXene suggested that the pure resin could not effectively prevent bacterial growth, while the inconspicuous inhibition zone in the group with 2 mg MXene might be due to the very low MXene load. The diameters of the inhibition rings gradually increased with increasing MXene content (Figures 4A, B) confirming the superior antibacterial effect of the incorporated MXene.

**FIGURE 5:**
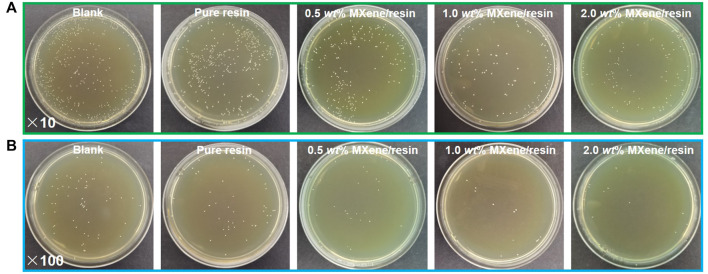
**(A)** Agar plates showing bacterial growth for varying dilutions of each solution containing *S. mutans* in contact with as-synthesized MXene/resin nanocomposites after incubation for 12 h. The dispersion containing *S. mutans* was diluted by **(A)** 10-fold and **(B)** 100-fold.

As mentioned above, the amount of MXene loaded in the dental resin played an important role in inhibiting bacterial growth. This study used *S. mutans* to test the antibacterial ability of the as-fabricated MXene/resin nanocomposites (Figure 5). The results showed gradual improvement in antibacterial activity with increased MXene loading. Notably, the MXene/resin nanocomposites showed excellent antibacterial activity for MXene loading above 0.5 wt%.

**FIGURE 6:**
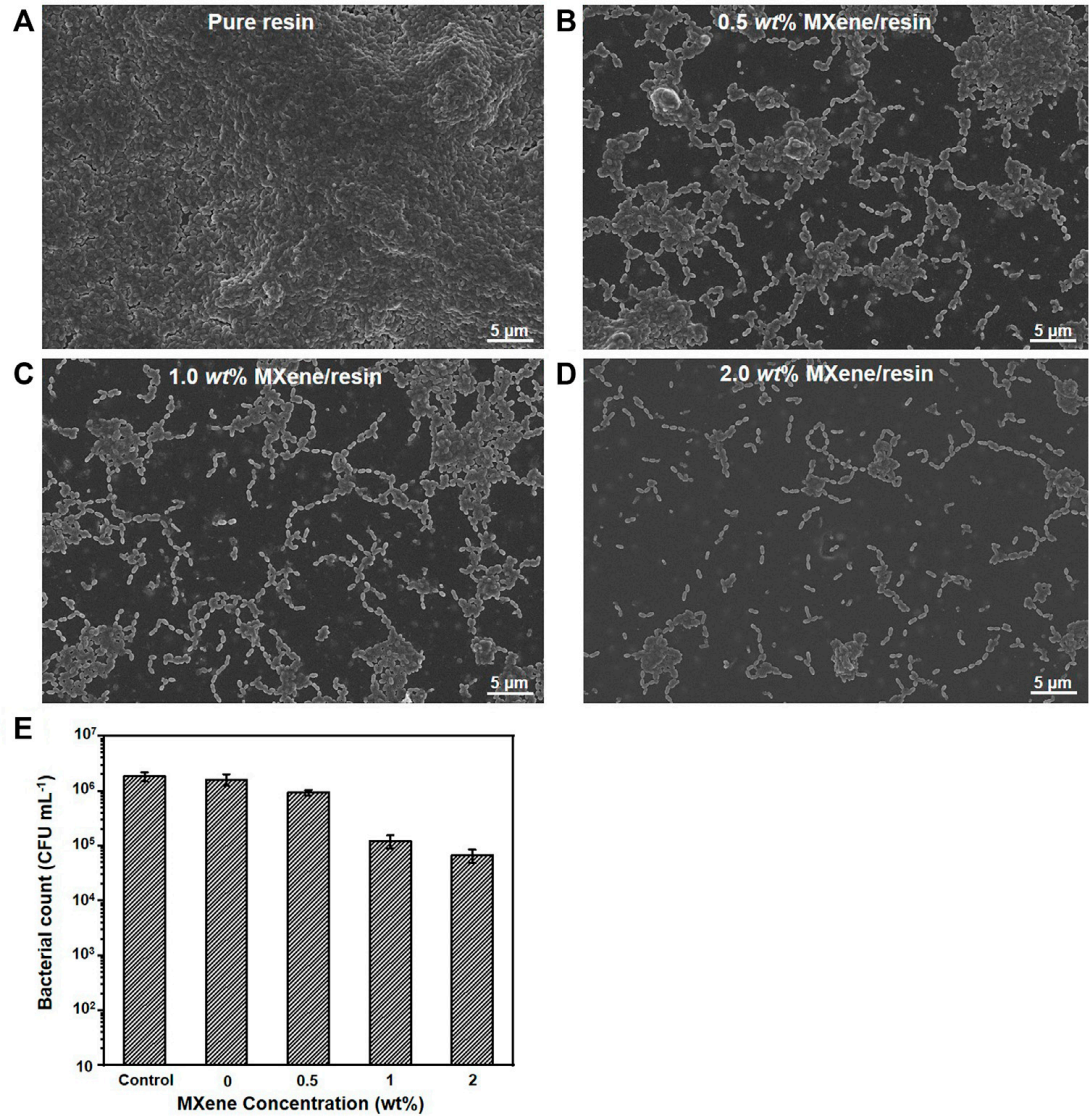
SEM images showing bacterial growth on the surface of as-synthesized MXene/resin nanocomposites: **(A)** pure resin, **(B)** 0.5 wt% MXene/resin, **(C)** 1.0 wt% MXene/resin, and **(D)** 2.0 wt% MXene/resin. **(E)** Bacterial count as a function of MXene concentration in the nanocomposite.

The superior antibacterial activity of the as-fabricated MXene/resin nanocomposites was also confirmed by SEM, as shown in Figure 6. Figures 6A–D show that as the MXene loading increased, fewer *S. mutans* bacteria were observed, and dispersed bacterial colonies were observed for MXene loading in the nanocomposite exceeding 1.0 wt%, consistent with the results shown in Figure 5. Moreover, Figure 6E shows significantly decreased *S. mutans* survival in MXene ranges from 0 (1.60 × 10^6^ CFU mL^−1^) to 0.5 wt% (9.29 × 10^5^ CFU mL^−1^), with slow reductions in the range of 0.5 wt% (9.29 × 10^5^ CFU mL^−1^) to 2.0 wt% (6.63×10^4^ mL^−1^), indicating that the MXene used in this study had highly efficient antibacterial behavior.

**FIGURE 7:**
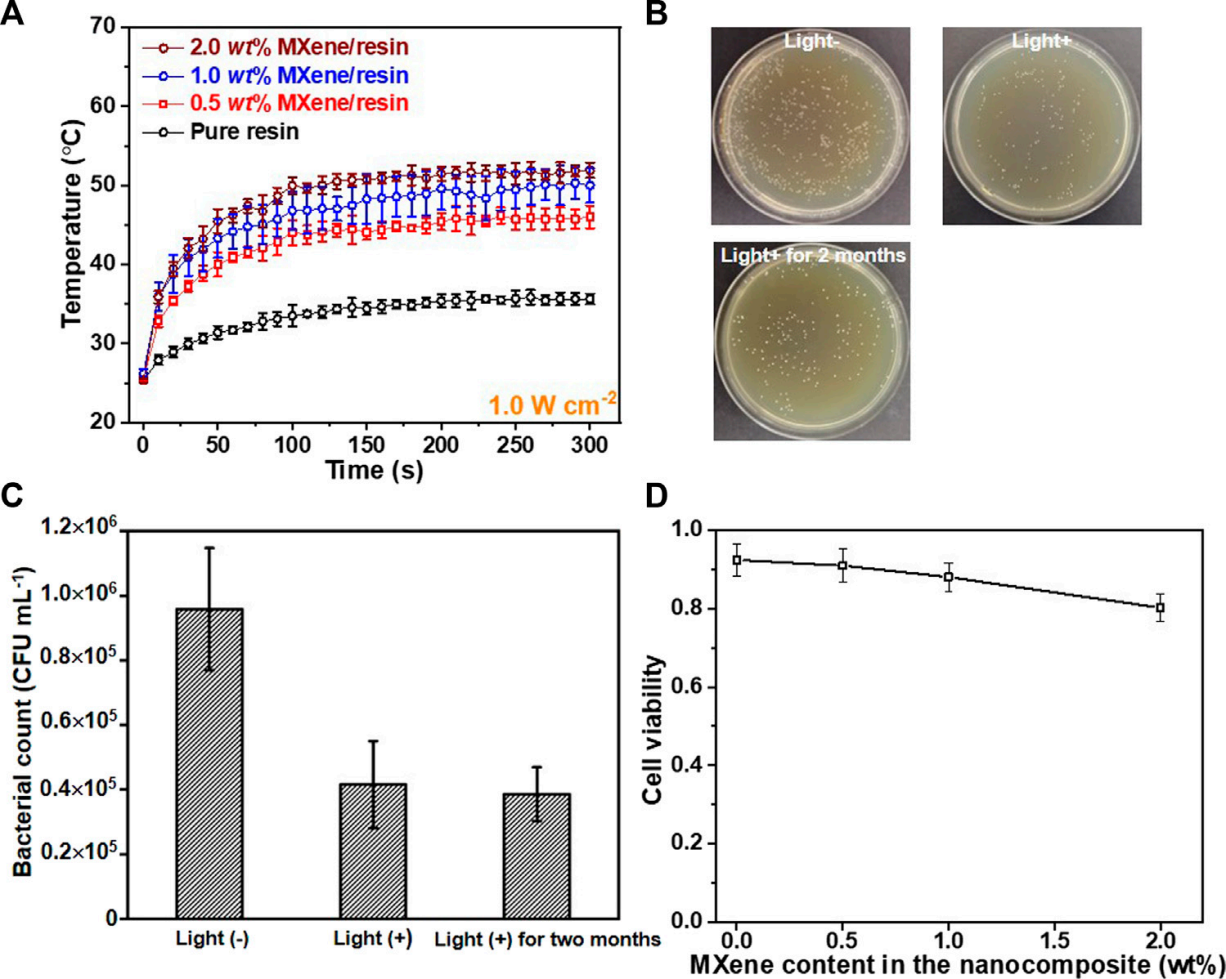
**(A)** Photothermal effect of as-synthesized MXene/resin nanocomposites. **(B)** Antibacterial activity of the 1.0 wt% MXene/resin nanocomposite in dark (light-), under natural light (light+), and light + for 2 months. **(C)** Corresponding trends in antibacterial activity and **(D)** cytotoxicity of the 1.0 *wt*% MXene/resin nanocomposite toward periodontal ligament fibroblasts.

In addition, due to the high photothermal effect of MXene (Huang et al., 2020b; Wang M. et al., 2021), the effects of the photo-to-heat conversion on antibacterial activity of the MXene/resin nanocomposites were also assessed after natural light illumination for 5 min (Figure 7). The photothermal effect of MXene in Figure 7A showed that with increasing MXene loading, the surface temperature of the nanocomposites gradually improved, with surface temperatures after illumination at 0.2 W cm^−2^ for 300 s of 35.7°C, 45.8°C, 50.0°C, and 51.9°C, respectively. The surface temperature (51.9°C) for 2.0 wt% MXene/resin was close to that of our previously reported MXene-based sponge (2.0 wt% MXene/sponge, 53.0°C) under the same conditions. Furthermore, evaluation of the photothermal effect of the MXene/resin nanocomposites on the antibacterial activity (Figures 7B, C) showed that distinctly enhanced inhibition of *S. mutans* growth upon exposure to natural light (120 mW cm^−2^, light+) for 24 h at 25°C compared to that in the dark (light-), indicating the further improvement of the MXene/resin nanocomposites under illumination due to the superior photothermal conversion of MXene. Surprisingly, the photothermal-assisted antibacterial property of the MXene/resin nanocomposites under natural light lasted at least 2 months without significant deterioration (Figures 7B, C), with *S. mutans* counts before and after 2 months of 4.16 × 10^4^ CFU mL^−1^ and 3.86 × 10^4^ CFU mL^−1^, respectively. More importantly, the MXene/resin nanocomposites showed negligible cytotoxicity toward normal oral cells, including periodontal ligament fibroblasts, even at an MXene loading of 2.0 wt%, demonstrating the promise of MXene/resin nanocomposites in practical dental applications.

## Conclusion

This study demonstrated the enhanced strength and superior antibacterial activity of MXene/resin nanocomposites, which are easily fabricated by solution blending and direct heating. The loading amount of the as-fabricated MXene/resin nanocomposites can be easily controlled by simply tuning the thin MXene Ti_3_C_2_T_x_ NSs. The TGA and DSC measurements show no apparent change in the thermal properties after the introduction of MXene. The results of the tensile and abrasion tests showed the improved strength and abrasion of the MXene/resin nanocomposites. The nanocomposites also exhibited significantly improved antibacterial behavior under natural light compared to that in the dark. More importantly, in the studied loading range of MXene, the as-fabricated MXene/resin nanocomposites did not severely damage normal cells in the oral environment, indicating the great promise of MXene/resin nanocomposites in dental applications. The facile fabrication, improved mechanical and abrasive properties, superior antibacterial activity, high photothermal efficiency, and low cytotoxicity of the MXene/resin nanocomposites suggest that they can shed light on the new designs of versatile MXene-based resins with multifunctional properties, such as photothermal/photodynamic therapy, multifunctional imaging-guided therapy, photothermal-assisted self-healing, self-healing/shape memory, *etc*., for practical dental applications.

## Data Availability

The original contributions presented in the study are included in the article/Supplementary material; further inquiries can be directed to the corresponding authors.
